# Revisiting the interleukin 17 family of cytokines in psoriasis: pathogenesis and potential targets for innovative therapies

**DOI:** 10.3389/fimmu.2023.1186455

**Published:** 2023-05-22

**Authors:** Nicolo Costantino Brembilla, Wolf-Henning Boehncke

**Affiliations:** ^1^ Divison of Dermatology and Venereology, Geneva University Hospitals, Geneva, Switzerland; ^2^ Department of Pathology and Immunology, Faculty of Medicine, University of Geneva, Geneva, Switzerland

**Keywords:** psoriasis, psoriatic arthritis, IL-17, IL-25, TH17 cells, TYK2

## Abstract

Psoriasis is a common chronic inflammatory skin disease, associated with substantial comorbidity. TH17 lymphocytes, differentiating under the influence of dendritic cell-derived IL-23, and mediating their effects *via* IL-17A, are believed to be central effector cells in psoriasis. This concept is underlined by the unprecedented efficacy of therapeutics targeting this pathogenetic axis. In recent years, numerous observations made it necessary to revisit and refine this simple “linear” pathogenetic model. It became evident that IL-23 independent cells exist that produce IL-17A, that IL-17 homologues may exhibit synergistic biological effects, and that the blockade of IL-17A alone is clinically less effective compared to the inhibition of several IL-17 homologues. In this review, we will summarize the current knowledge around IL-17A and its five currently known homologues, namely IL-17B, IL-17C, IL-17D, IL-17E (also known as IL-25) and IL-17F, in relation to skin inflammation in general and psoriasis in particular. We will also re-visit the above-mentioned observations and integrate them into a more comprehensive pathogenetic model. This may help to appreciate current as well as developing anti-psoriatic therapies and to prioritize the selection of future drugs’ mode(s) of action.

## Introduction

Psoriasis is a frequent, chronic, noncommunicable inflammatory skin disease, for which there is no clear cause or cure. Psoriasis affects all ethnicities and of all ages. The disease manifests as well-defined, red, scaly plaques, appearing with a chronic-recurrent course at preferential sites such as elbows, knees, and scalp (1). Individuals with psoriasis are at an increased risk of developing other chronic and serious diseases, including psoriatic arthritis, metabolic syndrome, cardiovascular diseases and depression (2, 3).

Psoriatic inflammation is triggered by environmental factors, such as infections (4). These initiate an innate immune response with dendritic cells playing a major role. A well-defined cascade starting with keratinocyte damage results in release of self-nucleic acids, which are subsequently complexed to antimicrobial peptides, and sensed by plasmacytoid dendritic cells (pDCs) (5). These produce IL-6 as well as transforming growth factor β, and induce differentiation of naïve T-lymphocytes towards a TH17 phenotype, which is subsequently maintained by IL-23, derived from the above-mentioned pDCs (6). TH17 cells are named after their key effector cytokines, namely different isoforms of IL-17 (7). These cytokines target keratinocytes, which respond by producing IL-17s themselves, but also neutrophil-attractant chemokines, thus constituting a positive feedback-loop (comprising dendritic cells and TH17-cells) and boosting inflammation further though recruitment of additional inflammatory cells, namely neutrophils (8). The presence of tissue-resident memory T-cells contributes to the chronicity of the disease and may explain the recurrent appearance of lesions at previously affected sites (9).

Taken together, the concept of a central pathogenetic axis evolved, starting with activated dendritic cells that produce IL-23 and thus favoring the development of TH17-cells, which exhibit their effector functions through IL-17s (10). Additional processes directly linked to this axis are leukocyte extravasation and migration as well as leukocyte activation, elements of which represent “drugable” targets (11). Indeed, all of these approaches have been or are currently being explored. However, most of the drugs currently used for targeted therapy of psoriasis block elements of the IL-23/TH17/IL-17 axis.

**Figure 1:**
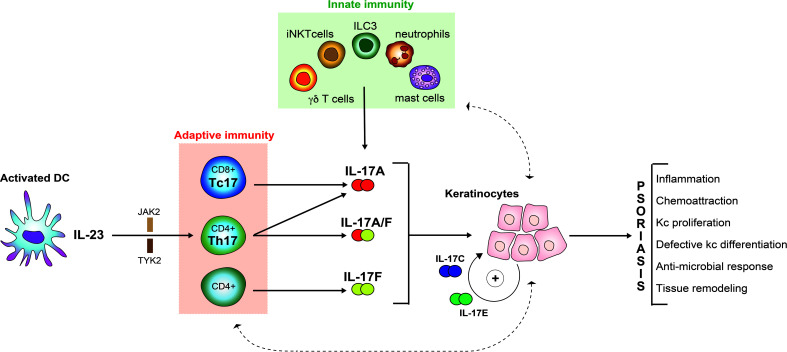
From linear to complex pathological model of psoriasis.

In this review, we will re-visit the above-mentioned observations and integrate them into a more comprehensive pathogenetic model (**Figure 1**). Subsequently, we will summarize the current knowledge around IL-17A and its five currently known homologues, namely IL-17B, IL-17C, IL-17D, IL-17E (also known as IL-25) and IL-17F, in relation to psoriasis with a focus on the most recent discoveries. In the last part of the review, we point towards the clinical implications of a more detailed understanding of IL-17 biology.

## From a “linear” to a “complex” pathogenetic model of psoriasis

The above-mentioned “linear” model of psoriasis has proven most helpful when it comes to understanding the impressive progress in terms of therapeutic efficacy in the field:

- Biologics targeting elements of the central pathogenetic axis exhibit unprecedented efficacy: In direct comparator studies, the conventional disease-modifying anti-rheumatic drug (DMARD) methotrexate was inferior to the TNF-α inhibitor adalimumab (PASI75 after 12 weeks: 25 versus 76%) (12); TNF-α inhibition by etanercept was inferior to IL-23/IL-12 inhibition by ustekinumab (PASI75 at week 12: 57 versus 74%) (13), which was inferior to the IL-17A inhibitor secukinumab (PASI90 at week 16: 58 versus 79%) (14).
- IL-17A inhibition was shown to exhibit a faster mode of onset compared to IL-23 inhibition (15), which is in line with the idea that blocking a “downstream” effector cytokine such as IL-17A should reduce psoriasis symptoms faster than blocking an “upstream” regulatory cytokine such as IL-23.
- In contrast, IL-23 inhibition has a particularly long-lasting therapeutic effect, as evidenced in randomized-withdrawal studies (16). This observation was initially interpreted as being due to the longer time needed to produce TH17 cells again. More recent studies suggest that this may also be related to the reduction in resident memory T cells in the skin (17).

Despite these conformities between model-based predictions and clinical observations, more recent findings make it necessary to revisit this simple “linear” model. One such finding is that IL-23 inhibition is insufficient to completely suppress IL-17A production. This is due to the fact that IL-17A production is not restricted to conventional CD4+ T helper lymphocytes (TH17 cells). Innate-like lymphoid cells, namely γδ T-cells, invariant natural killer T cells (iNKTs), mucosal-associated invariant T-cells (MALTs), as well as innate lymphoid cells (ILCs) are often skewed to type-17 profiles and may substantially contribute to IL-17A production in an IL-23 independent manner [reviewed in (18)]. Besides, cells other than lymphoid cells are also capable of producing IL-17A (19). Moreover, IL-17 homologues other than IL-17A have long been overlooked. This is particularly true for IL-17F, which is actually more abundant in lesional psoriatic skin when compared to IL-17A (20). As IL-17 homologues exhibit partially overlapping biological effects (see below), molecules were developed that block IL-17A as well as IL-17F. This approach yields synergistic effects *in-vitro* (21) and proved superior to selective IL-17A inhibition in a direct comparator clinical trial (22).

## The IL-17 cytokine family – an update

**Figure 2:**
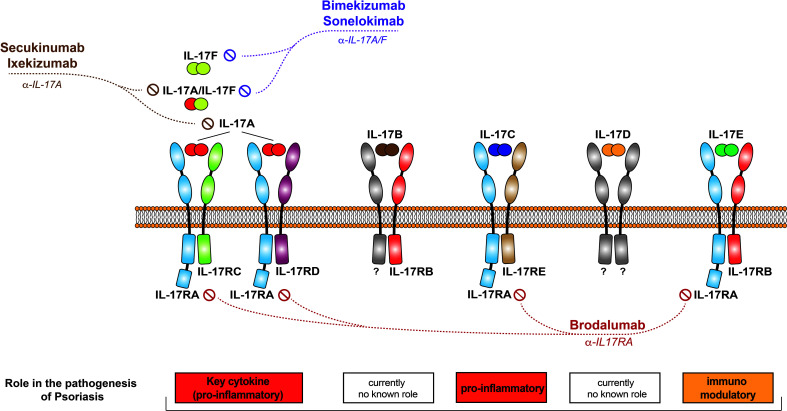
The IL-17 family of cytokines. Schematic representation of the different IL-17 cytokines, their receptors, and the currently available therapeutics that target them.

The IL-17 cytokine family consists of six members, named IL-17A to IL-17F (**Figure 2**). All members form functionally active homodimers. IL-17A also forms heterodimers with IL-17F. IL-17 cytokines signal through the IL-17 receptor family (IL17Rs), exclusively using the adaptor Act1 as the primary intermediate to activate downstream signaling events. A functionally active receptor comprises a combination of two of five homologous receptor subunits, IL-17RA to IL17RE (7).

### IL-17A

Described in 1993 and initially named CTLA-8, IL-17A is the most vigorously studied IL-17. It is produced by immune cells and targets primarily non-hematopoietic cells in barrier tissues, such as the skin, playing a central role in protective immune responses to extracellular pathogens and fungi.

IL-17A is classically considered to signal through the IL17RA-RC receptor, as do IL-17A/F heterodimers and IL-17F (7, 23). Recent data have however shown that these cytokines can also bind to a receptor composed of two IL17RC subunits, transducing signal in an IL-17RA-independent manner (24). The IL-17RA/RD heterodimer has also been reported as an alternative receptor for the IL-17A homodimer, but not for IL-17F/F and IL-17A/F (25, 26).

Activation of the IL17RA-RC heterodimer leads to the unique recruitment of the adaptor protein Act1 *via* homotypic SEFIR domain interaction. Act1 acts like a docking station for TRAF proteins, facilitating target gene transcription through the activation of NFκB, MAPK and C/EBP pathways. Additionally, activation of various RNA-binding proteins (such as HuR and Regnase-1) downstream the IL-17RA-RC receptors allows IL-17A to control post-transcriptional events, often resulting in increased target RNA stability (7, 27). Recent crystallography data point to the formation of a 2:2:2 hexameric signaling assembly composed of two units each of IL-17A, IL-17RA and IL-17RC. IL-17RA dimerization was shown to be functionally important, leading to the potentiation of IL-17-induced IL-36γ and CXCL1 mRNA expression in human keratinocytes (28).

IL-17A is secreted by IL-23-dependent Th17 cells in the skin. Additional cellular sources exist. 10 years ago, it was demonstrated that CD8+ T cells located in the epidermis of lesional psoriatic skin secrete IL-17A, possibly upon recognition of antigens presented by HLA-Cw6 (29). These so-called Tc17 cells exhibit a resident memory phenotype and express both PD-1 and IL-23R (30). Additionally, neutrophils, mast cells, and innate immune cells (MAILT, ILC3 and γδ T-cells) all are capable of producing IL-17A in an IL-23 independent manner (19, 31–33).

IL-17A has four major effects on mesenchymal and epithelial cells in the skin:

- establishing a pro-inflammatory loop geared towards activation of neutrophils and Th17 responses through induction of inflammatory mediators and chemoattractants such as IL-8, CCL20, and G-CSF
- promoting a cathelicidin- and defensin-dominated antimicrobial response
- de-regulating the keratinocyte differentiation program, leading to decreased expression of late differentiation molecules and loosening of the epidermal barrier
- promoting metalloproteinase-dependent tissue remodeling, facilitating the influx of newly recruited leukocytes

With regard to keratinocyte proliferation, recent publications show that IL-17A induces proliferation, either directly through activation of YAP-AREG signaling in keratinocytes, or indirectly *via* IL-19 and IL-24, produced in response to IL-17A in dermal stromal cells (34, 35).

Finally, new data suggest that IL-17A may alter immune regulatory responses. In psoriasis, regulatory T cells are dysfunctional, exhibiting reduced suppressive capacity and an exhausted phenotype (36, 37). The underlying mechanism comprises reduced TGF-β release and increased IFN-γ production. This skewing is reversed under therapy with IL-17A inhibitors (38). In addition, IL-17A (as well as IL17F and the IL-17A/F heterodimer) induces resistance to CD8-mediated suppression in CD4+ T cells primed with IL-17A, which is reversible *via* blocking IL-1β, IL-6, or STAT3 (39).

### IL-17F

L-17F shows the highest homology to IL-17A amongst all IL-17 family members. Besides homodimers, there exist IL-17A/F heterodimers as well. IL-17F signals *via* the canonical IL-17A receptor, composed of an IL17RA and -RC subunit. It has functions similar to IL-17A, namely with regard to an effective defense against muco-epithelial bacterial and fungal infections. IL-17F is generally considered less “potent” than IL-17A, with the IL-17A/F heterodimer exhibiting an intermediate potency (40).

Similar to IL-17A, IL-17F is expressed by immune cells of both innate and adaptive lineages, including Th17 cells, γδ T cells, and ILC3. IL-17F is believed to be generally co-expressed with IL-17A (the two genes are at the same locus), but this concept is being challenged by more recent data, analyzing emigrating cells from psoriasis samples at a single cell level. Such analyses show that most T17 cells express either IL-17A or IL-17F, with <10% of cells co-expressing both cytokines (36). Interestingly, most T cells in lesional psoriatic skin express IL-17F and not IL-17A. These cells exhibit a cytokine profile which differs from “conventional”, i.e. IL-17A-producing T cells. This may explain the clinical observation that simultaneous blockade of IL-17A and IL-17F resulted in successful treatment of patients who failed to respond to selective anti IL17A therapy before (41). Besides, IL-17F may also play a role in the recurrent course of psoriasis (42).

While the contribution of IL-17F in psoriasis is now accepted, its role in psoriatic arthritis remains under debate. To this end, expression of both IL-17A as well as IL-17F has been shown to be increased in synovial tissue and entheses of patients with psoriatic arthritis. While data from head-to-head comparator trials are not yet available, indirect evidence suggests comparable efficacies of selective IL-17A inhibition and blockade of IL-17A along with -F (43).

### IL-17C

IL-17C is only 23% homologous to IL-17A and, unlike the latter, is expressed by and acts on epithelial cells. Keratinocytes are the main producers of and responders to IL-17C in the skin. Signaling occurs *via* a receptor composed of the IL-17RA and IL-17RE subunits. IL-17Cs physiological role is to establish antimicrobial protective responses at barrier tissues.

It is now also widely accepted that IL-17C contributes to the psoriatic inflammation. It is increased in lesional skin, induces a gene expression pattern similar to that induced by IL-17A in keratinocytes *in vitro*, and is pathogenic in animal models *in vivo* (44–46).

Recent data, based on bioinformatic analysis of RNA seq experiments comparing non-lesional skin with the leading edge of evolving psoriatic plaques, identified IL-17C as a functional regulator of the initial psoriatic cytokine network, suggesting a role during the early stages of psoriatic inflammation, or the “priming” for plaque formation (47).

Of interest, the level of IL-17C in lesional skin was reduced to levels comparable to non-lesional skin upon therapy with an anti-IL17A/F inhibitor (48), and effective systemic therapy with methotrexate, ustekinumab, Secukinumab, or adalimumab were all associated with markedly decreased IL-17C concentrations in peripheral blood (49). These data suggest a potential role for IL-17C as a biomarker for therapeutic efficacy.

### IL-17B and IL-17D

IL-17B signals through a receptor that contains the IL-17RB subunit. While IL-17B is recognized for its role in cancer pathophysiology, it may not play a major role in the context of inflammation (50). Its concentration in psoriatic plaques is low (51).

Little is known about IL-17D, and its receptor has yet to be identified. IL-17D induces the production of pro-inflammatory cytokines and appears to play a regulatory role in anti-tumor and anti-viral responses (52). IL-17D levels are decreased in chronic plaque-type (51), but increased in palmoplantar pustulosis (53). It inhibits the expression of DDX5 helicase RNA in keratinocytes, promoting the production of membrane-bound IL-36R, and leading to the amplification of IL-36R-mediated skin inflammation. This newly identified pathway suggests a novel role of IL-17D in the control of skin inflammatory disorders, which needs to be further elucidated and studied in the context of pathology (54).

### IL-17E (also known as IL-25)

L-17E, also known as IL-25, shares only 6% homology with IL-17A. It signals through a heterodimeric receptor composed of the IL-17RA and IL-17RB subunits. IL-17E is produced by and acts on several cell types, including those of epithelial, mesenchymal, and hematopoietic origin.

Although primarily recognized as a cytokine that participates in type 2 cell responses (55), IL-17E may also play a role in the maintenance of skin inflammation at large (56). Keratocyte-derived IL-17E promotes the expression of pro-inflammatory cytokines in an autocrine manner, participates in the amplification of the psoriatic inflammatory network (57–59), and promotes recruitment of neutrophils in neutrophilic dermatoses (60).

IL-17E exhibits effects on keratinocytes that are distinct from those of IL-17A in as much as it induces cell motility and proliferation rather than antimicrobial responses. IL-17Es functions clearly go beyond those of a “simple” alarmin; it seems to play an important role in epidermal homeostasis (61).

Interestingly, serum levels of IL-17E could perhaps be used as biomarkers of psoriasis activity or severity, as particularly high levels are detectable in erythrodermic psoriasis (62).

## Therapeutic implications of a more detailed understanding of IL-17 biology

In recent years, IL-17 inhibition has become a mainstay of psoriasis treatment. Currently approved anti-psoriatic drugs interfering with IL-17 function comprise biologics and a JAK inhibitor, additional molecules are under development (11). These are (**Table 1**, **Figure 2**):

- Antibodies selectively blocking IL-17A (secukinumab, ixekizumab)
- Molecules blocking IL-17A and –F (bimekizumab, sonelokimab)
- Inhibitors of IL-17 signalling (brodalumab)

**Table 1:** Synopsis of anti-psoriatic drugs interfering with IL-17s. Only molecules with published positive phase III trial data are included.

Mode of action	Molecule	Approved for psoriasis therapy	Comment
Binding to IL-17A	Secukinumab	Yes	Fast mode of onset
Binding to IL-17A	Ixekizumab	Yes	Fast mode of onset
Binding to IL-17A and –F	Bimekizumab	Yes	• Mode of onset faster than anti IL-17As • more effective compared to secukinumab • candida infections more common compred to selective IL-17A inhibition
Binding to IL-17A and –F	Sonelokimab	No	Nanobody
Binding to the IL-17RA receptor	Brodalumab	Yes	Risk of relapse/rebound
Binding to IL-23 (p19 subunit)	Guselkumab	Yes	• Slower mode of onset compared to anti IL-17As • long lasting efficacy after withdrawal
Binding to IL-23 (p19 subunit)	Risankizumab	Yes	• Slower mode of onset compared to anti IL-17As • long lasting efficacy after withdrawal
Binding to IL-23 (p19 subunit)	Tildrakizumab	Yes	• Slower mode of onset compared to anti IL-17As • long lasting efficacy after withdrawal
JAK inhibitor	Tofacitinib	No	Less effective compared to antibody therapies
TYK2 inhibitor	Deucravacitinib	Yes	Less effective compared to antibody therapies

Besides, IL-23 inhibitors can be regarded as indirect IL-17 inhibitors, as this mode of action results in reduced production of IL-17A. All of these molecules exhibit distinct efficacy and safety profiles reflecting their respective mode of action.

Secukinumab and ixekizumab are monoclonal antibodies binding IL-17A selectively and directly. They are both highly effective, and “clear skin” became a feasible treatment goal in psoriasis with the approval of these drugs, as most patients achieved treatment responses superior to the previous treatment goal of a 75% reduction in the Psoriasis Area and Severity Index (PASI) (63). They also exhibit a particularly fast mode of onset, as expected by the fact that they block a key “effector” cytokine directly (see above). This is evidenced by superior efficacy data in direct comparator studies against IL-23 inhibitors during the induction phase of treatment (15, 64). As IL-17A plays an important role in mucosal immunity in general and protection against infections (namely by candida) in particular, it is not surprising that candida infections are a common adverse event of these drugs (65). Nevertheless, the interest in novel strategies to block IL-17A remains unbroken, as exemplified by clinical development programs using innovative molecules such as izokibep, a fusion protein exhibiting a particularly high affinity to IL-17A (66) or the oral IL-17 inhibitor DC-806 (67).

IL-17F is an IL-17 homologue who’s relevance in the pathogenesis of psoriasis has long been overlooked. Once it was realized that IL-17F is actually more abundant in lesional psoriatic skin (20), with partially overlapping functions (21), it became an obvious target for novel anti-psoriatic therapies. The monoclonal antibody bimekizumab has recently been approved for this indication. It binds IL-17A and IL-17F homodimers as well as the IL-17A/F heterodimer. As it blocks two strongly pro-inflammatory cytokines involved in the pathogenesis of psoriasis, a greater depth of clinical response was hypothesized (68). This hypothesis proved right, as bimekizumab showed superior efficacy in a direct comparator trial against secukinumab. But as IL-17F does not only contribute to psoriasis, but also to mucosal immunity, the blockade of IL-17F along with IL-17A results in an increased risk for candida infections (22). Another molecule binding to both IL-17A and IL-17F is the nanobody sonelokimab. The clinical trial data published so far document its efficacy in the treatment of psoriasis (69).

Finally, the monoclonal antibody brodalumab blocks the IL-17RA receptor and thus signaling of IL-17A, -C –E, and –F. As expected, this strategy is highly effective (70). The brodalumab clinical development program was abruptly interrupted for safety reasons (several cases of major adverse cardiovascular events in the brodalumab arm but not the placebo arm in one clinical study, resulting in a statistically significant safety signal) and subsequently resumed (brodalumab’s safety profile with regard to such major adverse cardiovascular events has recently been documented by an integrated safety analysis based on 5 clinical trials (71). Discontinuation of brodalumab treatment resulted in numerous relapses and even rebounds (72). This observation may be explained by the fact that a mediator may still be present (or even increased in concentration due to blockade of potential feedback loops), ready to signal through the receptor once it becomes available for interaction again. Meanwhile, brodalumab is approved for the treatment of psoriasis.

IL-17 inhibition may also be achieved indirectly *via* blocking IL-23 and consequently a reduction of sources of IL-17. There are currently three anti IL-23 antibodies approved for the treatment of psoriasis, namely guselkumab, risankizumab, and tildrakizumab. Their efficacy is comparable to the one of the IL-17 blocking antibodies (63, 73). However, as expected when blocking an upstream “regulatory” cytokine, clinical trial data tend to document longer time to onset of action in studies assessing approved dosing ranges of IL-23 inhibitors compared with studies assessing IL-17 inhibitors (74). However, speed of onset does not depend on a drug’s mode of action alone, but is also influenced by affinity and potency, as well as the selected clinical end point. Therefore, individual comparisons between one specific IL-23 inhibitor and one specific IL-17 inhibitor may yield different results.

Blocking the intracellular signal transduction of IL-23 at the level of the JAKs follows a similar reasoning. The pan JAK inhibitor tofacitinib as well as the specific TYK2 inhibitor deucravacitinib, have both shown clinical efficacy in clinical trials (75, 76). Deucravacitinib has meanwhile been approved for the treatment of psoriasis, while the manufacturing company of tofacitinib decided not to file for approval.

In this review, we focused on psoriasis. From a physician’s perspective, it is also important to which extent psoriatic comorbidities can either also be treated or represent potential contraindications for a given anti-psoriatic drug (77). This is also highlighted in the current S3 treatment guidelines (78). Clinically, the single most important comorbidity of psoriasis is psoriatic arthritis, affecting up to 30% of psoriasis patients (79). In the context of this review, it is therefore important to stress that all of the above-mentioned anti-psoriatic drugs are also effective in the treatment of psoriatic arthritis. IL-17A inhibitors are by now readily accepted by rheumatologists and recommended by expert groups such as EULAR (80) or GRAPPA (81). JAK inhibition is an established treatment strategy for psoriatic arthritis as well (82). And there are positive data from a phase II trial with the above-mentioned TYK2 inhibitor deucravacitinib (83). Finally, guselkumab was the first IL-23 inhibitor approved for psoriatic arthritis (84), exhibiting efficacy data comparable to IL-17A inhibitors (85).

In summary, our growing understanding of the IL-17 cytokine family helps us to better understand the pathophysiology of psoriasis as well as the distinct profiles of numerous drugs either already approved or in advanced stages of clinical development for this indication with regard to efficacy, mode of onset, and safety. To this end, it seems as if direct blockade of more than one IL-17 homologue potentially increases efficacy, but also characteristic safety signals (candida infections), while modulating the inflammatory milieu *via* interference with signal transduction may result in a lower clinical efficacy compared to direct blockade of IL-17. This may open the door towards development of systemic therapies for moderate rather than severe psoriasis as well as – finally – innovative topical therapies, as small molecules such as JAK inhibitors may be used in topical formulations. It remains to be seen to which extent IL-17 homologues other than –A and -F are valid targets for anti-psoriatic therapies. One company’s Il-17C program has recently been shut down (WHB, personal communication). And IL-17E/IL-25 as an important player in epithelial homeostasis at large might be more relevant as therapeutic target in other pathologies (56).

## Author contributions

W-HB and NB jointly selected the topic for the review, performed the literature review, wrote the manuscript, and designed the figure along with tables. All authors contributed to the article and approved the submitted version.
